# The Associations between Rapid Strength Development and Muscle Stiffness in Older Population

**DOI:** 10.3390/healthcare9010080

**Published:** 2021-01-15

**Authors:** Masatoshi Nakamura, Ryosuke Kiyono, Shigeru Sato, Kaoru Yahata, Taizan Fukaya, Satoru Nishishita, Andreas Konrad

**Affiliations:** 1 Institute for Human Movement and Medical Sciences, Niigata University of Health and Welfare, Niigata 950-3198, Japan; masatoshi-nakamura@nuhw.ac.jp (M.N.); hpm19005@nuhw.ac.jp (R.K.); hpm19006@nuhw.ac.jp (S.S.); hpm20011@nuhw.ac.jp (K.Y.); fukaya.taizan@gmail.com (T.F.); 2 Department of Physical Therapy, Niigata University of Health and Welfare, Niigata 950-3198, Japan; 3 Department of Rehabilitation, Kyoto Kujo Hospital, Kyoto 601-8453, Japan; 4 Institute of Rehabilitation Science, Tokuyukai Medical Corporation, Osaka 560-0054, Japan; satoru@rehalab.jpn.org; 5 Kansai Rehabilitation Hospital, Tokuyukai Medical Corporation, Osaka 560-0054, Japan; 6 Institute of Human Movement Science, Sport and Health, University of Graz, Graz A-8010, Austria

**Keywords:** rate of force development, shear elastic modulus, plantar flexor, ultrasound

## Abstract

Background: Previous studies suggest that the capacity for rapid force production of ankle plantar flexors is essential for the prevention of falls in the elderly. In healthy young adults, there were significant associations between rate of force development and muscle stiffness measured by shear wave elastography. However, there has been no study investigating the association of rate of force development with shear elastic modulus in older adults. Methods: The muscle strength and shear elastic modulus of the medial gastrocnemius muscle in both legs were measured in 17 elderly men and 10 elderly women (mean ± SD; 70.7 ± 4.1 years; 160.6 ± 8.0 cm; 58.7 ± 9.5 kg). We investigated the rate of force development of plantar flexors and shear elastic modulus of medial gastrocnemius muscle using by shear wave elastography. Results: Our results showed that there were no significant associations between normalized rate of force development and shear elastic modulus of medial gastrocnemius muscle. Conclusion: This suggests that the capacity of rapid force production could be related not to muscle stiffness of the medial gastrocnemius muscle, but to neuromuscular function in older individuals.

## 1. Introduction

Falls are a serious problem facing the elderly, and they can lead to fractures, hospitalization, and even death. Generally, one-third of community-dwelling elderly individuals aged over 75 years will experience at least one fall each year [1]. In addition, studies have shown that the decline in balance function in the older population can lead to an increased risk of falls [2,3]. Among lower limb muscles, the plantar flexors are especially important for postural control, mobility, and other motor functions [4,5]. A previous study showed that older female fallers had 23% lower rapid force production of plantar flexors rather than non-fallers [6]. In addition, Ema et al. investigated the association between balance function and muscle strength or rapid force production of plantar flexors [7]. The results of this study showed that the capacity for rapid force production rather than maximum muscle strength of ankle plantar flexors was essential for balance function. These results suggest that the capacity for rapid force production of ankle plantar flexors is essential for the prevention of falls in the elderly.

Ando et al. (2019) investigated the association of maximum muscle strength or rate of force development (RFD) and cross-sectional area (CSA) or shear elastic modulus of the medial gastrocnemius (MG) in healthy young adults [8]. These results showed that there was a significant association between maximum muscle strength and CSA, whereas there was no significant association between maximum muscle strength and the shear elastic modulus of the MG. On the other hand, there was a significant positive association between RFD and the shear elastic modulus of the MG. Regarding age-related changes in the shear elastic modulus of the MG, Nakamura et al. reported no significant difference between young and older women [9]. Thus, it can be assumed a significant association between rapid force production and the shear elastic modulus in older adults. To the best of our knowledge, however, there has been no study investigating the association of rapid force production with shear elastic modulus in older adults. Considering the aforementioned studies [8,10], a higher shear elastic modulus of the MG could contribute to both higher capacity of rapid force production and to improved balance function. Therefore, the aim of this study was to investigate the association between rapid force production and shear elastic modulus of the MG in older adults.

## 2. Materials and Methods

### 2.1. Participants

The muscle strength and shear elastic modulus of the MG in both legs were measured in 17 elderly men and 10 elderly women. Their baseline characteristics (mean ± SD) were 70.7 ± 4.1 years; 160.6 ± 8.0 cm; and 58.7 ± 9.5 kg. Participants who lived in the neighborhood of the laboratory who met the inclusion criteria were recruited by telephone. The inclusion criteria were age > 65 years, residing in the community, and the ability to walk independently (with or without a cane). The exclusion criteria were cognitive impairment, severe cardiac or musculoskeletal disorders, previous diagnosis of pulmonary disease, and hearing impairment. Some participants (25.9%) were engaging in regular exercise at the time of the experiment. All participants were fully informed of the procedures and purpose of the study, and all provided written informed consent.

The sample size required for a correlation analysis (effect size = 0.5 [large], α error = 0.05, and power = 0.80) was calculated using a G* power 3.1 software (Heinrich Heine University, Düsseldorf, Germany) based on a previous study (Ando et al., 2019), and hence, more than 26 participants were required in this study.

### 2.2. Assessment of Maximal Voluntary Isometric Contraction (MVIC) Torque and Rate of Force Development (RFD)

**Figure 1:**
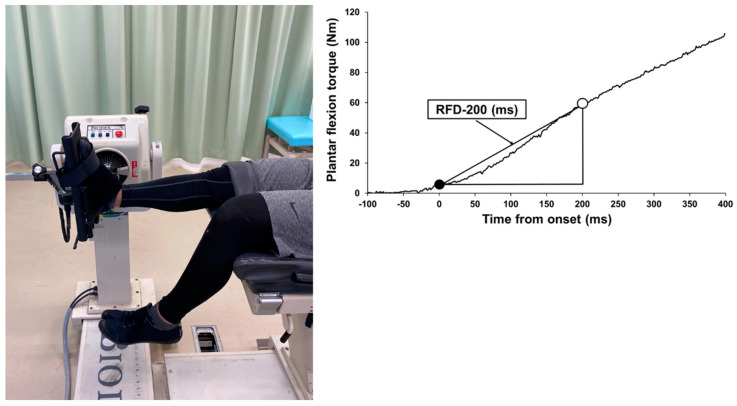
The experimental set-up for plantar flexor strength measurement and typical example of rate of force development (RFD) at 200 ms (with filtering correction).

Participants were seated on the isokinetic dynamometer (Biodex System 3.0, Biodex Medical Systems Inc., Shirley, NY, USA) chair at a 0° knee angle (anatomical position), with adjustable belts fixed over the trunk and pelvis. They were then reclined (55° hip angle) to prevent tension at the back of the knee (Figure 1). After several warm-up submaximal plantar flexion contractions, the participants were instructed to perform plantar flexion as fast and as hard as possible while maintaining plantar flexion, for about 3 s. The trials were conducted three times more than 30 s rest between each trial. Torque signals were recorded on a computer through an A/D converter operating at 1 kHz (PowerLab16/35, AD Instruments, Bella Vista, Australia). Torque signals were low-pass filtered at 15 Hz using a fourth-order zero-phase lag Butterworth filter [7,11]. The peak value of each torque signal was taken as the MVIC torque. Thereafter, the onset of plantar flexion was defined as the point at which torque increased 2 SD above baseline and did not fall below baseline throughout the contraction (3 s). The RFD was defined as the slope of the filtered time-torque curve over time intervals of 0–50, 0–100, 0–150, and 0–200 ms from the onset of plantar flexion [7,11]. In addition, to exclude the effect of the MVIC torque, the RFD was normalized to MVIC torque (normalized RFD).

### 2.3. Assessment of the Shear Elastic Modulus of the MG

We measured the shear elastic modulus of the MG using ultrasonic shear wave elastography (SWE) (Aplio 500, Toshiba Medical Systems, Tochigi, Japan) using a 5–14 MHz linear probe using the same sitting position as was used for muscle strength measurement. Then, we measured the shear elastic modulus of the MG at 30% of the lower leg length from the popliteal crease to the lateral malleolus near the point of the maximal cross-sectional area of the lower leg [12,13]. We obtained elastographic images in duplicate of the state of the long-axis image of the MG and obtained ultrasound images using custom, image analysis software (MSI Analyzer version 5.0, Rehabilitation Science Research Institute, Japan). We drew the quadrangular region of interest (ROI) as large as possible within the color-coded area of the elastographic images while accounting for the artifact from the aponeurosis. The software automatically calculated the average value of Young’s modulus in the quadrangular ROI. Based on previous studies [12,13], we calculated the shear elastic modulus by dividing the obtained Young’s modulus by 3.

### 2.4. Statistical Analysis

We used SPSS software (version 24.0; SPSS Japan Inc., Tokyo, Japan) for statistical analysis. The normality of all variables was confirmed using Shapiro–Wilk tests. We calculated partial correlations between the shear elastic modulus of the MG and RFD in all time intervals adjusted for age, body mass index, and gender. We assumed differences to be statistically significant at an alpha level of *p* < 0.05. We have presented descriptive data as means ± standard deviation.

## 3. Results

Normalized RFD and shear elastic modulus values are shown in Table 1. There were no significant associations between shear elastic modulus of the MG and normalized RFD.

## 4. Discussion

In this study, we investigated the association between rapid force production and the shear elastic modulus of the MG in older adults. Our results showed that there were no significant associations between RFD in all time intervals and the shear elastic modulus of the MG. Although there has already been a study investigating the association between RFD and shear elastic modulus in young adults, this is the first study to investigate the association between RFD and shear elastic modulus in older adults.

Regarding rapid force production and stiffness of series elastic components in the muscle-tendon unit, Ando et al. reported a significant association between the shear elastic modulus of the MG and RFD in all time intervals in young adults [8]. Moreover, Waugh et al. investigated the association between rapid force production and tendon stiffness in prepubertal children and healthy adults and showed that there was a significant association between them [14]. Considering these previous studies, a higher stiffness of series elastic components in the muscle-tendon unit could contribute to higher capacity of rapid force production, and we hypothesized that there could be a significant association between the shear elastic modulus of MG and normalized RFD in older adults. However, our results were inconsistent with our hypothesis.

The previous study by Ando et al. (2019) showed the shear modulus of MG correlated significantly with normalized RFD at all time intervals (*r* = 0.460–0.496) in young adults [8]. Although there was a significant correlation, the magnitude of these correlations can be defined as medium rather than large. Therefore, besides possible age differences, it is possible that no significant correlations were found between shear elastic modulus of MG and RFD in this study with older adults. The other reason why there was no significant association between the shear elastic modulus of the MG and normalized RFD at all time intervals might be related to the shear elastic moduli of the lateral gastrocnemius muscle (LG) and soleus rather than the MG. The previous study reported that there was no significant age-related change in the shear elastic modulus of the MG [9], whereas the shear elastic modulus of the LG was lower in older adults than young adults [15]. In addition, previous studies showed that aches and tendon stiffness in older adults were significantly lower than in young adults [16,17]. In young adults, Kubo et al. reported that there was no significant association between muscle stiffness and tendon stiffness [18]. The relationship between muscle stiffness and tendon stiffness in older adults is unclear; tendon stiffness could be related to rapid force production in older adults. Moreover, previous studies have shown that RFD was related to neuromuscular function, measured by muscle activity [19,20]. Therefore, RFD might be related to neuromuscular function rather than to muscle stiffness in older adults. Therefore, future studies are needed to investigate the association of rapid force production and shear elastic modulus of other muscles and neuromuscular function in older adults.

Balance function has been shown to be significantly related to the rapid force production of plantar flexors [7]. In addition, previous studies showed that RFD increased after resistance training intervention [11,21]. On the other hand, Akagi et al. reported that there was no significant change in shear elastic modulus after six weeks of resistance training [22]. Moreover, Ema et al. investigated eight-week high-speed calf-raise training for community-dwelling older adults. These results showed that there were significant increases in balance function and RFD, and these changes could be related to neuromuscular adaptation [23]. These previous studies taken together with our results indicate that a change in tendon stiffness and/or neuromuscular adaptation rather than a change in muscle stiffness could be necessary to improve balance function in older adults. Therefore, further studies are needed to investigate the effect of resistance training on muscle stiffness, RFD, and balance function in community-dwelling older adults.

There were some limitations in this study. Previous studies showed changes in muscle composition (i.e., intramuscular adipose tissue and muscle density) with aging in vivo [24,25,26], which can affect shear elastic modulus values. Thus, future studies are needed to take the different muscle compositions into account while assessing shear elastic modulus. In addition, we did not measure the balance function and hence, the relationship between balance function and RFD or shear elastic modulus remains unclear. Therefore, in future studies, it is needed to investigate the balance function and relate it to shear elastic modulus or RFD.

## 5. Conclusions

Our results showed that there were no significant associations between the shear elastic modulus of the MG and normalized RFD at all time intervals in older individuals. This suggests that the capacity of rapid force production could be related, not to muscle stiffness of the MG, but to neuromuscular function in older individuals.

## Figures and Tables

**Table 1:** The outcome variables and partial correlations between shear elastic modulus of medial gastrocnemius and rate of force development (RFD) at all time intervals.

Parameters	Mean ± SD	Association with Shear Elastic Modulus	
		*R* Value	*p* Value
Shear elastic modulus (kPa)	9.6 ± 8.7	-	-
Normalized RFD-50 (%MVC/s)	256.8 ± 111.4	−0.042	0.768
Normalized RFD-100 (%MVC/s)	267.2 ± 107.8	−0.029	0.843
Normalized RFD-150 (%MVC/s)	258.9 ± 86.4	0.024	0.868
Normalized RFD-200 (%MVC/s)	246.5 ± 71.0	0.019	0.892

## Data Availability

All data generated or analyzed during this study are included in this published article.
